# Nutrient Composition and Physical Properties of Two Orange Seed Varieties

**DOI:** 10.1155/2021/6415620

**Published:** 2021-10-11

**Authors:** Joseph Adubofuor, Yaw Gyau Akyereko, Vida Batsa, Osborn-Jnr Doetser Apeku, Isaac Amoah, Charles Diako

**Affiliations:** ^1^Department of Food Science and Technology, Faculty of Biosciences, Kwame Nkrumah University of Science and Technology, Kumasi, Ghana; ^2^Department of Food and Post-Harvest Technology, Faculty of Applied Science and Technology, Koforidua Technical University, Koforidua, Ghana; ^3^Department of Biochemistry and Biotechnology, Faculty of Biosciences, Kwame Nkrumah University of Science and Technology, Kumasi, Ghana; ^4^School of Food and Advanced Technology, Massey University, Auckland, New Zealand

## Abstract

Orange is mainly consumed as fresh fruit, concentrated juice, or thin dried slices, while the seeds are usually discarded by consumers and orange juice processing companies. This study was carried out to determine the physical, frictional, nutritional, and antinutritional properties of the Late Valencia and Red Blood orange seeds. The proximate composition, mineral profile, antinutrient content, and physical and frictional properties of two orange seed varieties were determined using standard methods. The mean length, width, thickness, geometric, and arithmetic mean diameter of the Late Valencia and Red Blood seeds were 14.66, 8.45, 5.05, 8.47, and 9.39 and 13.74, 7.51, 4.99, 7.97, and 8.75 mm, respectively. An angle of repose 39.62° and 38.62°, coefficient of friction of 0.63 and 0.61 on wood, 0.33 each on mild steel, and 0.41 and 0.43 on Teflon were recorded, respectively, for the Late Valencia and Red Blood orange seed varieties. Seeds from Late Valencia and Red Blood orange variety contained 547.39 mg/100 g and 693.87 mg/100 g of oxalate, respectively. Proximate and vitamin C of the orange seeds analyzed indicated that the protein (4.18%), fat (57.45%), fiber (6.06%), energy (640.66 kcal/100 g), and vitamin C (63 mg/100 g) content in the Late Valencia were significantly higher (*p* < 0.05) than the Red Blood orange (3.61%, 55.77%, 5.49%, 85 kcal/100 g, and 54 mg/100 g) correspondingly. The high fat content of the orange seeds makes them potential source of oil for both food and nonfood product applications. Ca, P, and K were predominantly found in the two orange seed varieties. Physical and frictional properties obtained from this work could aid in the design of equipment for harvesting, processing, transporting, separating, packaging, and storage of orange seeds from Late Valencia and Red Blood orange varieties. Further research is required to determine the suitability of orange seed flours for value-added products.

## 1. Introduction

Citrus species belongs to the family Rutaceae and is an annual plant widely distributed in the Mediterranean countries of the Middle East and Southern Europe but also thrives in other warm climates [1] including Ghana. In terms of production capacity, Ghana produced 775,705 tonnes of oranges in 2019 [2]. Orange is consumed mainly as fresh fruit, concentrated juice, or thin dried slices [1]. The seeds remain one of the by-products from the fruit consumption and industrial processing. Orange seeds are perceived to be of low economic value. Consequently, the seeds are mostly discarded creating environmental nuisance as it results in the generation of greenhouse gases and additionally lead to economic losses by the food industry [3]. Varieties of orange include common oranges (*Citrus sinensis*), navel oranges, blood oranges, and acidless oranges [4, 5], and these varieties are distinguished based on their unique taste, colour, texture, and juice yielding characteristics.

In a recent review of published studies on the bioactive and nutritional properties of orange seeds, fatty acids, phytosterols, tocopherols, fibers, minerals, and vitamins were reported as the predominant nutrients [6–8]. Orange seeds contain oil ranging from 34.92 to 41.66% [7, 9], and the oil is rich in unsaturated and essential fatty acids including oleic and linoleic acids [9]. Additionally, the seeds contain bioactive compounds such as carotenoids and flavonoids [6, 10]. These compounds have potential health-promoting effects as they act as antioxidants [11] and impair the activities of free radicals. That notwithstanding, the presence of antinutrients which at high concentrations interferes the absorption of certain essential micro- and macronutrients in food is present in orange seed. Some examples of these antinutrients include oxalate, phytate, saponins, nitrate, and cyanide [12, 13].

Physical properties of seeds from plants including hemp [14], quinoa [15], and lentil [16] have been analyzed. Determination of the physical properties of fruits and seeds is essential in the designing of equipment for sowing, harvesting, transporting, storing, packaging, and processing seeds prior to extraction of oil [17–19]. There is currently a paucity of data on the physical properties and antinutritional properties of orange seed. Examination of the physical properties of orange seeds and profiling of its antinutritional and nutrient content is essential to inform industry players on critical parameters to consider in the design of equipments for the processing of orange seeds. This could enhance the potential valorisation prospect of orange seeds for high value product application. The objective of this study was to determine the nutrient composition, antinutrient, and physical properties of seeds and fruit of orange, specifically Late Valencia and Red Blood varieties.

## 2. Materials and Methods

### 2.1. Sample Collection and Preparation

Late Valencia and Red Blood orange samples were obtained from CSIR-Crop Research Institute-Fumesua, Kumasi, and Krofofrom, a suburb in Kumasi, respectively. The oranges were grouped into three categories: small, medium, and large based on size.

The orange fruits were cut to remove the seeds. The seeds were washed thoroughly and prepared for use by spreading them uniformly on trays after which the trays were placed in solar dryers until a constant weight of seeds was obtained. The physical and frictional properties of the seeds were then determined. The seeds were dehulled, milled to fine powder using a laboratory mill (42823CO, Hamburg), and stored in zip lock bags at room temperature prior to antinutrient composition, proximate, mineral, and vitamin C analyses. Antinutrients determined were oxalates and alkaloids.

### 2.2. Physical Properties of Orange Fruits

#### 2.2.1. Weights of Fruits

The weights of the oranges were determined using a digital electronic balance (ML204/01, Switzerland).

#### 2.2.2. Heights and Circumference of Fruits

Hundred oranges were randomly selected from thousand available oranges. The heights of the hundred oranges were determined using a plastic metre rule of 100 cm long and a rope, and the circumference of the fruits was determined using a vernier caliper (500-196-20, USA) of accuracy of 0.01 mm.

### 2.3. Physical Properties of Orange Seeds

#### 2.3.1. Axial Dimensions

To determine the axial dimensions, length (*L*), width (*W*), and thickness (*T*) of the orange seeds, 100 seeds were randomly picked, and the three linear dimensions of the seeds were determined using vernier calliper (500-196-20, USA) of accuracy of 0.01 mm [17].

#### 2.3.2. Arithmetic and Geometric Diameter

The arithmetic (Da) and geometric diameters (Dg) of the seeds were calculated using the relations of [13].

#### 2.3.3. Thousand Seed Weight

The thousand seed weight was determined by the method of Mohsenin [17].

#### 2.3.4. Densities and Porosity

Bulk density (*ρ*_*t*_) of the orange seeds was determined following the method of Mohsenin [17]. True density (*ρ*_*t*_) was calculated by weighing 20 g of the seeds. The weighed seeds were counted (*n*) and multiplied by the volume, *V* = (*π*/6) *D*_*g*_^3^ [20]. The weighed seeds (*M*) were divided by the resultant product of the volume and number of seeds *M*/*nV*, [21].

Porosity was calculated from bulk density and true density using the relation by Mohsenin [17].

#### 2.3.5. Sphericity Aspect Ratio, Surface Area, and Volume [22–24]

(1) $$\begin{array}{c} \text{Sphericity Ø}=\frac{{\left(\text{LWT}\right)}^{1/3}}{L}, \end{array}$$

(2) $$\begin{array}{c} \text{Aspect ratio Ra}\%=\left(\frac{W}{L}\right)*100, \end{array}$$

(3) $$\begin{array}{c} \text{Surface area }S=\pi \;{\left(\text{Dg}\right)}^{2}, \end{array}$$

(4) $$\begin{array}{c} \text{Volume }V=\frac{\pi }{6\left({\text{Dg}}^{3}\right)}. \end{array}$$

### 2.4. Frictional Properties

Frictional property analyses involved the determination of coefficient of static friction and angle of repose.

#### 2.4.1. Coefficient of Static Friction

The coefficient of static friction was measured on three test surfaces, namely, wood, Teflon, and mild steel. The device used is made up of the test surface, an inclined plane, and an adjustable protractor. The test surface was fixed on the plane and adjusted to lie horizontally flat on the base. The adjustable protractor was then fixed between the plane and the base and set to 0°. The short cylindrical container opened at both ends was placed on the test surface and filled with the seeds. The open-ended container was also pulled up a bit so that it was no more in contact with the test surface leaving only the seeds still in contact with the test surface. The inclined plane was raised gently, and the angle of inclination at which the samples started sliding was read off the protractor and recorded *Θ*. The tangent of the angle (tan *Θ*) was then recorded as the coefficient of static friction (*μ*). The relation is *μ* = tan *Θ* [25].

#### 2.4.2. Angle of Repose

The angle of repose was determined using a cylindrical container opened at both ends and a wooden circular base of known diameter (*D*). The cylinder was placed on the wooden circular base, filled with the seeds, and raised slowly until a cone of seeds formed on the base. The height (*H*) of the cone formed was measured using a rule and a rope. The angle of repose was calculated using the following equation [25]:

(5) $$\begin{array}{c} \Theta =\tan^{-1}\;\left(\frac{2H}{D}\right). \end{array}$$

### 2.5. Proximate Analyses of Orange Seeds

The moisture, ash, protein, fat, and carbohydrate contents of the orange seeds were determined according to the standard official methods of AOAC [26]. Theenergy content was calculated using the Atwater factor by multiplyingthe protein content by 4 kcal/g, carbohydrate content by 4 kcal/g, andthe fat content by 9 kcal/g. The energy content was calculated using the Atwater factor by multiplying the protein content by 4 kcal/g, carbohydrate content by 4 kcal/g, and the fat content by 9 kcal/g.

### 2.6. Mineral Composition Determination

Minerals were determined using the official method of the AOAC [27]. Sodium (Na) and potassium (K) were determined using the standard flame emission photometer (PFP 7). NaCl and KCl were used as the standards. Calcium (Ca), phosphorus (P), and iron (Fe) were determined using Atomic Absorption Spectrophotometer (S.P-300, Canada). The absorbance of P, Ca, and Fe was read at 770 nm, 540 nm, and 520 nm, respectively. All values were expressed in mg/100 g.

### 2.7. Vitamin C (Ascorbic Acid) Determination

The method of AOAC [26] was used in the determination of vitamin C content.

### 2.8. Antinutrient Determination of Orange Seeds

The oxalate and alkaloid contents of the orange seeds were determined. The method described by Day and Underwood [28] was used in determining oxalate content whereas alkaloids was analyzed according to the method described by Harborne [29]. The laboratory analyses were done in duplicate.

### 2.9. Statistical Analysis

Data from the research was analyzed using IBM SPSS statistic version 20. Significant difference between the means was determined by the Student *t*-test using the independent variance test. Classification tree analysis was done using XLSTAT (Addinsoft, France). Significant difference was established at *p* < 0.05.

## 3. Results and Discussion

### 3.1. Physical Properties of Late Valencia and Red Blood Orange Fruits

The mean mass of the small, medium, and large oranges of the two varieties ranged from 188.76 to 304.25 g (Table 1).

The mass of the Late Valencia and Red Blood orange varieties differed significantly (*p* < 0.05) between the large and small fruits. Generally, the Late Valencia orange variety had higher weight than the Red Blood oranges. The mean mass of tangerine samples (Clementine variety) analyzed by Rashidi and Keshavarzpour [30] was found to be 101.2 g which was lower than the varieties of oranges analyzed in this study. The mean masses of the small- and medium-sized fruits of both varieties in the present study were in the range of values determined by Topuz et al. [31] which ranged from 175.71 to 228.92 g. The mean circumference and mean height of the large and medium fruits of the Late Valencia and Red Blood orange varieties were not significantly different (*p* > 0.05). For the small-sized fruits, the mean circumference of the Late Valencia orange variety was significantly lower (*p* < 0.05) than that of the Red Blood orange variety. Work done by Topuz et al. [31] gave values ranging from 6.92 to 8.26 cm in terms of height (length), and a similar range of values was obtained for the large- and medium-sized fruits of both varieties in this study. Also, comparing work done by Sharifi et al. [32] on Tompson variety of orange to this study, the heights of the large-sized Late Valencia and Red Blood orange fruits were lower than the large-sized Tompson fruits which had mean value of 9.04 cm. The same trend followed when the mean heights of the medium- and small-sized Late Valencia and Red Blood fruits were compared with the mean heights of the medium- and small-sized Tompson fruits of mean values 8.40 and 7.79 cm, respectively. Consumers prefer fruits of equal weight and uniform shape. Mass grading of fruit can reduce packaging and transportation costs and may also provide an optimum packaging configuration [33]. More fruits with lower weights and heights can be filled in packaging containers, hence making transportation easier than fruits with larger weights and heights. Sizing by weighing mechanism is recommended for the irregular shape products such as citrus fruits [34].

### 3.2. Physical Properties of Late Valencia and Red Blood Orange Seeds

Information on the physical properties of seeds is needed in the design and adjustment of equipment for cleaning, handling, storing, and converting agricultural materials into food, feed, and fodder. In theoretical calculations, agricultural seeds are assumed to be sphere or ellipse because of their irregular shapes [35]. Most of the research done on the physical properties of seeds are based on the seeds as a function of moisture content. According to Aviara et al. [36], the moisture dependent characteristics of physical properties have an effect on the adjustment and performance of agricultural product processing machine. The design of seed processing equipment without considering its engineering specifications may yield poor results [37].

#### 3.2.1. Axial Dimensions (Length, Width, and Thickness)

Table 2 shows the results on the physical properties of the two different orange seed varieties. The shape of a seed is either exploited singly or with other properties to determine the free flowing characteristic or bridging tendencies of seeds in many separators. Shape and size of seed refer to the properties of the seed which determine how much space it occupies and within limits can be described in terms of the length, width, and thickness. Axial dimension consists of length, width, and thickness and plays an important role in determining aperture sizes used in grain handling machines as established by Mohsenin [17]. The mean length, width, and thickness of the Late Valencia orange seeds were 14.66, 8.45, and 5.05 mm, respectively, while the corresponding mean length, width, and thickness of Red Blood orange seeds were 13.75, 7.51 and 4.99 mm, respectively. Statistical analysis showed no significant differences (*p* > 0.05) in the mean length and thickness of Late Valencia and Red Blood orange varieties, but the width varied significantly (*p* < 0.05). This implies that the design of the aperture sizes in the handling machine of these two varieties of oranges will be different when the width is taken into consideration. Shape is exploited singly or together with other characteristics to determine the free flowing or bridging tendencies of seed mass in many separators used in cleaning [38]. Davies [37] determined engineering properties of three varieties of melon seeds as potentials for development of melon processing machines as a function of their moisture content. It was noted that the highest axial dimension was observed for the seeds of *C. vulgaris* with mean length, width, and thickness of 14.50, 8.47, and 2.49 mm, respectively. The mean length and width of the seeds of *C. Vulgaris* melon variety were similar to the seeds of the Late Valencia orange variety, but the mean thickness was different. Gupta and Das [39] determined the average length, width, and thickness of sunflower seeds at 6.2% moisture content d.b to be 9.52, 5.12, and 3.27 mm, respectively, which is lower than the values obtained for both Late Valencia and Red Blood oranges in this study. Davies [37] reported that the separation of biomaterials as a unit operation is dependent on the axial dimensions.

#### 3.2.2. Geometric and Arithmetic Diameters of Late Valencia and Red Blood Orange Seeds

From Table 2, the mean arithmetic and geometric diameters of the Late Valencia orange seeds were 9.39 and 8.47 mm, respectively, while that of the Red Blood orange were 8.75 and 7.97 mm, respectively. The geometric and arithmetic mean diameters of these two varieties at 95% confidence interval were not significantly different (*p* > 0.05). The geometric mean diameter of the axial dimension is also useful in the estimation of the projected area of the particle moving in a turbulent or near turbulent region of an air stream. This projected area of the particle indicates the movement or pattern of the particle in air as well as ease in the removal of extraneous materials from the particle during cleaning by pneumatic means. This means that, statistically, the behavioural characteristics of Late Valencia orange seeds in their movement in air and also the removal of the extraneous materials are not different from Red Blood orange seeds. The arithmetic diameters are also used to design sieving and grading machines by measuring their perpendicular diameters. The arithmetic and geometric mean diameter determined by Lorestani et al. [40] had values of 13.43 and 13.42 mm, respectively, for castor seed. These values are higher than the arithmetic and geometric diameters of the seeds of the orange varieties determined in this study. This range of values is slightly lower but close to values obtained for the two orange varieties determined in this study. The arithmetic and geometric mean diameters are dependent on the dimensions of the seeds. The dimensions of the seeds are important in the design of sieve separators and in calculating power during the seed milling process. Higher arithmetic and geometric mean diameters denote that the seeds have bigger dimensions; thus for the design of sieve separators, the sizes of the separators may be wider and bigger. Also, in the design of processing equipments, seeds with high arithmetic and geometric diameters will have wider aperture sizes than seeds with lower arithmetic and geometric mean diameters.

#### 3.2.3. Volume, Sphericity, and Aspect Ratio of Late Valencia and Red Blood Orange Seeds

The Late Valencia orange variety significantly had higher volume (325.21 mm^3^) and aspect ratio (58.84%) than the volume (268.62 mm^3^) and aspect ratio (55.60%) of the Red Blood orange variety (Table 2). However, the average sphericity of the seeds of both varieties of oranges was not significantly different (*p* > 0.05). The variation in the volume and aspect ratio of the two orange seed varieties can be attributed to climatic conditions (rainfall, humidity, wind), genetic composition of the plant or varietal differences, nutrient availability, position of seed in the inflorescence, and size of seed used for planting [41, 42].

The volume of a seed is the amount of three-dimensional space occupied by the seed. The volume of a seed is dependent on the linear dimensions, moisture content, surface area, and temperature. The volume to the surface area of the Late Valencia and Red Blood is 0.45 and 0.42, respectively. Any particle that has a smaller ratio of volume per unit surface has better conditions for rapid heat transfer [34]; thus, the Late Valencia seeds consume less time and energy during the drying processes.

The higher the value of sphericity of a seed, the closer the shape of seed approaches that of a sphere [43]. Sphericity is a degree of irregularity for an incompletely spherical shape of a solid grain relative to the same volume of same materials with a complete sphere [44]. The average sphericities of the Late Valencia and Red Blood varieties of orange seeds determined in this study were 0.59 and 0.59, respectively (Table 2) and were not significantly different (*p* > 0.05). The higher the value, the higher the seed approaches a sphere shape and the greater ability of the seeds to roll. The sphericity of a seed is critical for hopper, separator, and conveyor design [34].

Aspect ratio is an indicator of a tendency toward a particular shape [45]. The aspect ratio of the Late Valencia and Red Blood orange seeds was found to be 58.84 and 55.60%, respectively (Table 2). Late Valencia was significantly higher (*p* < 0.05) than Red Blood.

#### 3.2.4. Thousand Seed Weight and Surface Area of Late Valencia and Red Blood Orange Seeds

The measurement of thousand seed weight is an indicator of the grain size, which can vary relative to the growing conditions and maturity, even for the same variety of a given crop or fruit. This parameter is used to design seed sorters, screen holes, and containers for the agricultural produce. The thousand seed weight of agricultural products is exploited in the design of cleaning equipment using aerodynamic forces [46]. Moreover, the knowledge about 1000 seed weight is useful for the design of equipment related to aeration, drying, storage, and transport [47]. From Table 2, the average thousand seed weight of the Late Valencia was 153.58 g, which was not significantly different (*p* > 0.05) from 145.74 g for the Red Blood orange.

Surface area is the surface of the outer surface of an object. Surface area depends on the linear dimensions of solids and on their moisture content. In this study, from Table 2, the average surface area of the Late Valencia seeds was 713.77 mm^2^ which was significantly higher (*p* < 0.05) than the Red Blood orange seeds of value 629.97 mm^2^. Surface area depends on linear dimensions of seeds. Late Valencia orange seeds in this study had higher linear dimensions and also higher 1000-seed weight, thus, the higher surface area of the seeds. Higher surface area enhances the drying of the seeds since large surface of the seed is exposed to the drying air.

#### 3.2.5. Densities and Porosity of Late Valencia and Red Blood Orange Seeds

The bulk density gives an idea of the storage space required for a known quantity of a particular agricultural produce, for example, seeds. The bulk density of some grains increases with an increasing moisture content whereas it decreases for some other grains. From Table 2, the mean bulk densities of the seeds of Late Valencia and Red Blood investigated in this study were 365.08 and 394.39 kg/m^3^. These values were significantly different (*p* < 0.05). Higher bulk density implies that the seeds had a higher mass and a small volume; thus, more seeds can be filled into a small volume container. The Red Blood orange seeds had a higher bulk density than Late Valencia orange seeds; thus, in the filling of the seeds in two containers of similar volume, more Red Blood orange seeds can be filled into the container than the Late Valencia seeds.

Bulk density is also an important parameter because it determines the capacity of storage and transport. For storage containers, greater number of seeds of high bulk densities can be filled than seeds with lower bulk densities. Bulk density is also essential for the design of equipment for drying, aeration, and storage systems as this property affects the resistance to air flow of the mass systems [20]. Information on the true density will help in the design of equipment related to drying, storage, and also for transportation [47]. True density is also an important parameter for designing storage and transport systems. The mean true densities of the seeds of Late Valencia and Red Blood were 510.04 and 566.69 kg/m^3^, respectively, as shown in Table 2. These values were significantly different (*p* < 0.05). True density can affect the rate of heat and mass transfer of moisture during aeration and drying processes [47]. True density of grains decreases linearly with increasing moisture content. This implies that higher moisture contents of the seeds depict lower true densities. The porosity depends on true and bulk density of material. Porosity allows fluids to flow through a mass of particles referred to as a packed bed. Beds with low porosity are more resistant to fluid flow and thus are more difficult to dry, heat, or cool. This characteristic can be used to separate the seeds from other heavier foreign materials. It must be noted that porosity of the mass of seeds determines the resistance to airflow during aeration and drying process [46].

From Table 2, the mean porosities of Late Valencia and Red Blood were 28.42 and 30.40%, respectively, and there was no significant difference (*p* > 0.05) between them. Porosity of Late Valencia and Red Blood oranges was found to be lower than that of Tompson variety determined in grades of small, medium, and large as 51.20, 49.39, and 44.64%, respectively [32]. The porosity, which is the percentage of airspace in particular solids, affects the resistance to airflow through bulk solids and air flow resistance, in turn, affects the performance of aeration systems used to control the temperature of stored bulk solid [32].

**Figure 1:**
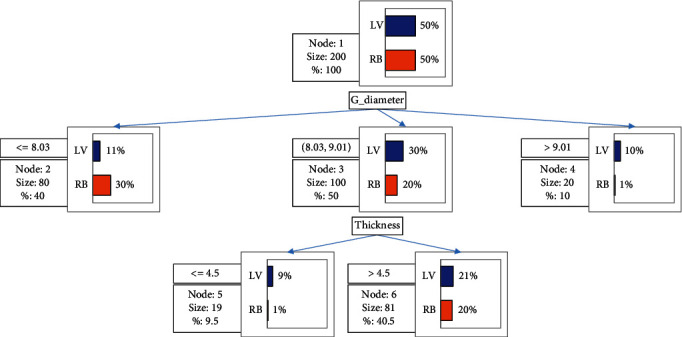
Classification tree showing the important characteristics (geometric diameter and thickness) that separate Red Blood (RB) and Late Valencia (LV) orange seeds.

The classification tree results (Figure 1) show that the geometric diameter (G-diameter) and thickness were the most important physical properties that descriminated between the Late Valencia and Blood Orange seed varieties. The first point of difference was the geometric diameter. The Late Valencia orange seeds were characterized by comparatively larger geometric diameter with the majority between 8.03 and 9.01 cm. In contrast, more of the Red Blood orange seeds had geometric means below 8.03 cm. Apart from this key difference based on the geometric diameter, the thickness of the orange seeds was the next distinguishing property. The Red Blood seeds had uniform thickness as most of the seeds were more than 4.5 cm in thickness compared to the Late Valencia seeds which had more seeds below 4.5 cm. These two parameters could potentially have implications for the design of equipments for handling, transporting, and processing of orange seeds.

### 3.3. Proximate Composition of Late Valencia and Red Blood Orange Seeds

The proximate composition and energy content of seeds from two orange varieties (Late Valencia and Blood orange) are presented in Table 3. The moisture contents of Late Valencia and Blood orange seeds were 3.14% and 3.54%, respectively, and were quite very low. There were significant differences (*p* < 0.05) between the moisture content of the two orange varieties determined. There is a greater potential of longer shelf life for the seeds due to the low moisture content. The low moisture content of the seeds gives a good suggestion on the suitability of the seeds for processing (milling, mixing, and oil extraction). The low moisture content of the flour would enhance its storage stability by preventing mould growth and reducing moisture dependent biochemical reactions [48].

The two orange varieties had a higher content of fat with Late Valencia having fat content of 57.45% which was not significantly different (*p* ≥ 0.05) from that recorded for Blood orange as 55.77%. The values obtained for the two varieties were not so much varied from 54.20% as reported by Samia El-Safy et al. [8] and Akpata and Akubor [49]. Egbuonu and Osuji [50] reported the fat content of *Citrus sinensis* (sweet orange) seeds to be 11.08%. The differences could be attributed to production factors, climate conditions, handling, and storage as stated by Steven [51].

The high oil content of the two orange seed varieties in this study makes them potential source for commercial vegetable oil extraction. Although, Late Valencia has the highest percentage of fat or oil but it could not be economically feasible as a lucrative business due to the low number of seeds produced per fruit (0-6 seeds) [52], and therefore, Blood orange seeds may be the best option for maximum and economical extraction of oil from orange seeds because of the large quantity of seeds produced per fruit (>6 seeds) [53].

The ash content of the Late Valencia and Red Blood orange seeds was found to be 2.44% and 2.48%, respectively. The values recorded were very close to 2.50% as reported by Samia El-Safy et al. [8] and Akpata and Akubor [49]. There was no significant difference (*p* ≥ 0.05) between the ash content of the two orange varieties. The percentage ash of a sample gives an idea about the inorganic content of the sample which the mineral content could be obtained. Samples with high percentages of ash contents are expected to have high concentrations of various mineral elements, which are expected to speed up metabolic processes and improve growth and development [54]. In view of this, the incorporation of either of the two varieties into any feed or food product for animals or human beings would aid in reducing micronutrient deficiency in plants and animals.

The analysis also revealed 4.18% and 3.61% as the protein content for Late Valencia and Red Blood orange seeds, respectively. Although, the values obtained for the two varieties were close to the 3.06% and 3.1% as reported by Samia El-Safy et al. [8] and Akpata and Akubor [49], respectively. Egbuonu and Osuji [50] reported a value of 6.77% for sweet orange (*Citrus sinensis*) seeds which is higher than the value obtained in this study. There was a significant difference (*p* < 0.05) between the protein contents of the seeds of both varieties. Abbas Ali et al. [55] explained that the difference in protein contents in seeds is affected greatly by the analytical method used for the assay. The protein content of the seeds as compared to other seeds, orange seeds would be considered as one of the high protein-containing seeds. The protein content of the seeds makes them a good source of commercial protein and hence its usage in fertilizer and animal feed formulation [56]. It is shown in Table 3 that Late Valencia seeds had higher amount of crude fiber (6.06%) than Red Blood orange (5.49%). The two orange varieties recorded higher crude fiber values than that obtained by Egbuonu and Osuji [50] in *Citrus sinensis* (2.98%) orange seeds. The percentage composition of crude fiber for the Late Valencia was significantly different (*p* ≥ 0.05) from that of the Red Blood orange. The variation could be due to genetic modification, production factors, climatic conditions, maturity stage, handling, and storage [51]. The carbohydrate contents of Late Valencia and Red Blood orange seeds were, respectively, 26.73% and 29.11%, and there was a significant difference (*p* < 0.05) between them. The value recorded for Blood orange was close to 28.5% as reported by Akpata and Akubor [49] but was very much varied from 34.74% as reported by Samia El-Safy et al. [8]. The carbohydrate content of Late Valencia shows a very wide variation between 34.74% and 28.5% as reported by both Samia El-Safy et al. [8] and Akpata and Akubor [49], respectively. El-Adawy et al. [57] reported the carbohydrate content of orange seeds (balady variety) to be 26.41% and 31.85% for mandarin (*Citrus mitis*) seeds. The variation was explained by Abbas Ali et al. [55] as due to genetic modification, climatic conditions, differences in soil properties, and method of analysis used. The carbohydrate contents of the seeds were very high, and therefore, their incorporation into other low energy products to supplement the carbohydrate content of such foods in order to meet the carbohydrate requirement in food products will be very relevant. The energy contents of the two orange varieties were found to be 640.66 kcal/100 g and 632.85 kcal/100 g, respectively, for Late Valencia and Blood orange seeds. The energy contents were very high and were significantly different (*p* < 0.05).

### 3.4. Mineral Composition of Late Valencia and Red Blood Orange Seeds

The results of mineral composition of the two orange seed varieties (Late Valencia and Red Blood orange) studied are shown in Table 4. The analysis revealed the contents of potassium in Late Valencia and Red Blood orange seeds to be 22.80 mg/100 g and 25.20 mg/100 g, respectively.

There was a significant difference in the two varieties (*p* < 0.05). Umeta et al. [58] reported that variation in soil nutrient, soil texture, land topography, fertilizer application, manure, genetic modification, and variety of plant accounts for the differences in the mineral composition of a plant and its bearings (fruits and seeds). The high content of potassium in the two seeds reveals their potential usage in animal and human food products to aid in pH regulation, maintenance of cellular water balance, and protein and carbohydrate metabolism [59]. The Late Valencia orange seeds contained higher amount of sodium (13.46 mg/100 g) compared to Blood orange which was 11.76 mg/100 g. The content of sodium in the Late Valencia orange seeds was significantly higher (*p* < 0.05) than that of blood orange seeds. The variation could be attributed to the differences in the soil properties of the land, the variety of crop, seasonal changes, level of maturation, and fertilization as explained by Vollmann et al. [60]. Late Valencia and Red Blood orange seeds had 93.85 mg/100 g and 82.60 mg/100 g of calcium, respectively. The calcium content of Late Valencia was significantly higher than that of Red Blood orange (*p* < 0.05). These values were far higher about twice that obtained by Samia El-Safy et al. [8] who investigated nutritional profile of orange seed flours. The values were however lower than the calcium content obtained by El-Adawy et al. [57] who analyzed the mineral composition of citrus seed flour. The unexpected vast variation is as a result of genetic modification, soil type, soil nutrient, climatic changes, and other agricultural practices including fertilization, weed control, and land preparation [58]. Calcium is of much importance in infants and pregnant women; therefore, supplementation of orange seeds in human foods will contribute immensely to bone and teeth formation and excellent blood circulation in the body.

The concentration of phosphorus in Late Valencia and Blood orange seeds was, respectively, 33.86 mg/100 g and 27.74 mg/100 g. The values were far lower than that reported by Samia El-Safy et al. [8] in their research work conducted on Late Valencia orange seeds having higher concentration of phosphorus (390.70 mg/100 g). The iron contents in the two orange seeds varieties were, respectively, 0.78 mg/100 g and 0.87 mg/100 g for Late Valencia and Red Blood orange seeds. There was no significant difference between the iron content of the two orange seed varieties (*p* ≥ 0.05) but was lower than what Samia El-Safy et al. [8] reported as 6.40 mg/100 g. The unexpected variation is as a result of genetic modification, soil type, soil nutrient, climatic changes, and other cultural practices including fertilization, weed control, and land preparation [58].

### 3.5. Vitamin C Content of Late Valencia and Red Blood Orange Seeds

The vitamin C contents of the two orange seed varieties were found to be 63 mg/100 g and 54 mg/100 g for Late Valencia and Red Blood orange, respectively, as shown in Table 4. There was a significant difference (*p* < 0.05) between the recorded values for the two orange seed varieties. Oranges are generally perceived to be high in vitamin C. The content of ascorbic acid in plants varies greatly, depending on genotypic differences, preharvest climatic conditions and cultural practices, maturity and harvesting methods, and postharvest handling procedures [61], but the most significant determinant of vitamin C content in foods is how the food is stored and prepared since vitamin C is easily oxidized leading to the eventual oxidation by oxygen in the atmosphere. The high vitamin C content is very surprising. The high concentration of vitamin C in the seeds encourages the utilization of the seeds in food system because vitamin C is required to prevent deficiency and stave off scurvy [62].

### 3.6. Antinutrient Composition of Late Valencia and Red Blood Orange Seeds

#### 3.6.1. Oxalate

Results of antinutrient (oxalate and alkaloid) content of seeds from the Late Valencia and Red Blood orange varieties are shown in Table 4. The oxalate contents of the Late Valencia and Red Blood orange variety seeds were 547.39 and 693.87 mg/100 g, respectively. The values recorded for both seeds were not significantly (*p* > 0.05) different. These values were relatively higher compared to findings by other researchers on the oxalate content of other oil containing seeds from other plants, and this could be ascribed to genotypic differences, growing environment, and harvesting stage of the plant or dissimilarities in the methods of extraction [63, 64]. Akande et al. [65] reported 410 mg/100 g and 490 mg/100 g of oxalates in large seeded and small seeded varieties of castor seeds, respectively. Oxalate is noted for its ability to chelate minerals including calcium forming calcium-oxalate during the digestion of food. Deposition of calcium-oxalate precipitates in the kidney promotes formation of kidney stones [66]. The maximum tolerable limit of oxalate is 0.25 mg/100 g [67]. This implies that beyond this level, the negative effects of oxalate in the human body including limited mineral bioavailability (Ca, Na, K), kidney stones (Ca-oxalate), peptic indigestion, and severe gut lining irritations will be experienced [65, 68]. The values obtained for the orange seeds in this study are exceedingly higher than the toxic level. The high content of oxalates in food materials can however be reduced if it is precooked before processing or eating [69, 70], and this can be recommended for the reduction of the oxalate content of the orange seeds.

#### 3.6.2. Alkaloids

The values for the alkaloid content recorded in this present study were 89.97 and 59.85 mg/100 g for Late Valencia and Red Blood orange seeds, respectively, as shown in Table 5. These values were lower than alkaloid content values reported for sesame seeds by other researchers as well as oxalate contents recorded in this same study. Alkaloids also tend to have toxic effects at a certain level apart from their general known bitterness due to the function they may specifically perform in protecting seeds from predators. Inuwa et al. [67] reports this toxic level as 20 mg/100 g. Diets containing alkaloids beyond this level will result in effects such as undesirable electrochemical transmissions produced as a result of the action of alkaloids on the nervous system, paralysis, and rapid heartbeats and in severe cases may lead to death, as well as symptoms of neurological disorder [68]. However, McDonald et al. [71] reported that the safe level for alkaloids in animal feed is 60 mg/100 g. The level in the Red Blood variety is almost equal to this value and can therefore be used safely in animal feed. Alkaloids in plant materials can however be removed completely by soaking in warm water at 60°C for 60 min [72].

### 3.7. Frictional Properties of Late Valencia and Red Blood Orange Seeds

#### 3.7.1. Angle of Repose

The values of angle of repose recorded in this study were 39.62 and 38.62° for Late Valencia and Red Blood varieties, respectively, as shown in Table 2. The moisture content levels at which these determinations were carried out were 3.14% and 3.54% for the Late Valencia and Red Blood varieties, respectively (Table 3). Differences in moisture content levels of granular materials affect their angle of repose. A higher moisture content corresponds to higher angles of repose with other factors such as shape and texture also coming into play [73]. On the contrary, Late Valencia with a lower moisture content has a higher angle of repose. This may however be attributed to varietal differences and different seed textures. There was however no significant difference (*p* > 0.05) between the angle of repose of both seeds. This suggests that the same equipment, storage, transportation, or handling systems can be used for both varieties of seeds. These values recorded are comparable to results published for wheat (36.7°), millet (35.79°), and rice (34.82°) with moisture contents approximately 5% by Aremu et al. [74].

#### 3.7.2. Coefficient of Static Friction

The coefficients of static friction for orange seeds from the Late Valencia and Red Blood varieties on three test surfaces, namely, wood, mild steel, and Teflon, are shown in Table 5.

The coefficient of friction on wood (0.63) for Late Valencia was slightly above that for Red Blood (0.61) whereas on Teflon, that of Red Blood was higher (0.43) as against Late Valencia (0.41).

Coefficient of static friction (just like angle of repose) is also affected by the moisture content of the agricultural raw material or seed and the surface characteristics of the test material. More rough surfaces will offer higher resistance to sliding of granular materials and hence a higher coefficient of static friction. Smoother surfaces offer less resistance, and so coefficients are smaller. This accounts for the higher recorded values on wood (0.63 and 0.61) and the least on steel (0.33). The behaviour of the two seeds on the wood, mild steel, and Teflon was not different significantly (*p* > 0.05). The highest coefficients were recorded on wood and the lowest on mild steel for both seeds. This trend agrees with previous findings as wood mostly has the highest coefficients and steel the least whenever these three surfaces are used [75, 76]. Values of coefficient of static friction are also used to determine the flowability of ensiled materials in silos and outlet chutes and to specify construction materials for such handling systems and equipment. The flowability of both seed varieties on the same surface would be virtually the same but different for each seed on the three surfaces. To specify a material for the construction of a system or equipment to handle, these seeds will therefore require the material offering less resistance to flow. Significant difference (*p* < 0.05) was found between the values of coefficient of static friction for each variety of seed on all the three surfaces, and the results from this study indicate that the seeds will flow best on mild steel surface, and hence, it will be appropriate to use mild steel as a construction material for equipment to be used in handling or processing both seeds as coefficient of friction was the same for both.

## 4. Conclusion

Orange seeds from Late Valencia and Red Blood orange varieties contain high amount of fat, fiber, energy, vitamin C, potassium, calcium, phosphorus, oxalate, and alkaloids. The average width of the Late Valencia seeds is significantly higher than that of Red Blood seeds, but the mean length and thickness are not different. The arithmetic, geometric mean diameters, average thousand seed weight, and average porosities of the orange varieties are not significantly different. However, the mean volume, surface area, and aspect ratio of the Late Valencia orange seeds are significantly higher than the Red Blood orange seeds. The average true and bulk densities of the Red Blood orange seeds are significantly higher than the Late Valencia orange seeds. The angle of repose and coefficient of static friction of the two orange seeds suggest that the same equipment or systems can be used to handle the two different seeds. Flowability of the seeds will therefore be considered as moderate and will need no intervention in discharge from storage systems or during transportation. Coefficient of friction values on wood is highest for each variety of seed followed by values on Teflon and the lowest on mild steel, which maybe considered as the most appropriate material surface for the design and construction of equipment to process the seeds.

## Figures and Tables

**Table 1:** Physical properties of Late Valencia and Red Blood orange fruits.

Size	Parameter	Late Valencia	Red Blood
Large	Circumference (cm)	6.95 ± 0.22^a^	6.87 ± 0.28^a^
	Height (cm)	7.45 ± 0.43^a^	7.17 ± 0.22^a^
	Weight (g)	304.25 ± 31.97^a^	260 ± 27.32^b^
Medium	Circumference (cm)	6.58 ± 0.23^c^	6.66 ± 0.22^c^
	Height (cm)	6.81 ± 0.20^c^	6.66 ± 0.16^c^
	Weight (g)	247.44 ± 17.35^c^	220.92 ± 22.11^c^
Small	Circumference (cm)	6.06 ± 0.32^d^	6.36 ± 0.19^e^
	Height (cm)	6.22 ± 0.23^d^	6.24 ± 0.21^d^
	Weight (g)	203.22 ± 13.74^d^	188.76 ± 15.64^e^

Mean values with the same superscripts in a row are not significantly different (*p* > 0.05).

**Table 2:** Physical properties of seeds of Late Valencia and Red Blood oranges.

Parameters	Late Valencia	Red Blood
Length (mm)	14.66 ± 1.86^a^	13.75 ± 1.67^a^
Width (mm)	8.45 ± 1.67^a^	7.51 ± 0.89^b^
Thickness (mm)	5.05 ± 1.14^a^	4.99 ± 0.62^a^
Arithmetic diameter (mm)	9.39 ± 0.76^a^	8.75 ± 0.58^a^
Geometric diameter (mm)	8.47 ± 0.69^a^	7.97 ± 0.53^a^
Volume (mm^3^)	325.21 ± 86.06^a^	268.62 ± 52.50^b^
Sphericity	0.59 ± 0.07^a^	0.59 ± 0.06^a^
Aspect ratio (%)	58.84 ± 15.52^a^	55.60 ± 10.35^b^
1000 seed weight (g)	153.58 ± 0.48^a^	145.74 ± 0.15^a^
Surface area (mm^2^)	713.77 ± 120.64^a^	629.97 ± 82.65^b^
Bulk density (kg/m^3^)	365.08 ± 3.27^a^	394.39 ± 4.51^b^
True density (kg/m^3^)	510.04 ± 4.92^a^	566.69 ± 6.92^b^
Angle of repose (^o^)	39.62 ± 1.25^a^	38.62 ± 2.99^a^

Mean values with the same superscripts in a row are not significantly different (*p* > 0.05).

**Table 3:** Proximate composition and energy content of seeds from two orange varieties on dry basis.

Parameter (%)	Late Valencia	Blood orange
Protein	4.18 ± 0.22^a^	3.61 ± 0.13^b^
Ash	2.44 ± 0.05^a^	2.48 ± 0.49^a^
Moisture	3.14 ± 0.08^a^	3.54 ± 0.07^b^
Fiber	6.06 ± 0.17^a^	5.49 ± 0.40^b^
Fat	57.45 ± 0.17^a^	55.77 ± 0.37^a^
Carbohydrate	26.73 ± 0.49^a^	29.11 ± 0.47^a^
Energy (kcal/100 g)	640.66 ± 0.94^a^	632.85 ± 0.75^b^

Mean values in the same row with different superscripts are significantly different (*p* < 0.05).

**Table 4:** Mineral, vitamin C, and antinutrient composition of orange seeds from two varieties.

Parameter (mg/100 g)	Late Valencia	Blood orange
Potassium (K)	22.80 ± 0.06^a^	25.20 ± 0.06^b^
Sodium (Na)	13.46 ± 0.12^a^	11.76 ± 0.18^b^
Calcium (Ca)	93.85 ± 0.49^a^	82.60 ± 0.42^b^
Phosphorus (P)	33.86 ± 0.13^a^	27.74 ± 0.50^b^
Iron (Fe)	0.78 ± 0.02^a^	0.87 ± 0.04^a^
Vitamin C	63.00 ± 1.39^a^	54.00 ± 0.13^b^
Oxalate	547.39 ± 60.73^a^	693.87 ± 31.77^a^
Alkaloids	89.97 ± 0.007^a^	59.85 ± 14.28^a^

Mean values in the same row with different superscripts are significantly different (*p* < 0.05).

**Table 5:** Coefficient of static friction of orange seeds on different surfaces.

Test surface	Late Valencia	Red Blood
Wood	0.63 ± 0.17^a,c^	0.61 ± 0.38^a,c^
Mild steel	0.33 ± 0.12^a,a^	0.33 ± 0.12^a,a^
Teflon	0.41 ± 0.12^a,b^	0.43 ± 0.17^a,b^

First letter(s) of superscripts compare(s) means in a row while second letter(s) compare(s) means in a column. Different superscripts in a column are significantly different (*p* < 0.05).

## Data Availability

The data that support the findings of this study are available from the corresponding author (Y. G Akyereko) upon reasonable request.
